# Self-diagnosing electromagnetic hypersensitivity—A case study

**DOI:** 10.3389/fpubh.2025.1535513

**Published:** 2025-03-12

**Authors:** David Ashton

**Affiliations:** Independent Researcher, Doncaster, United Kingdom

**Keywords:** electromagnetic hypersensitivity (EHS), RF radiation, world health organization, diagnosis, electric and magnetic fields (EMF), extremely low frequency (ELF), dirty electricity

## Introduction

‘*Exclude the impossible and what is left, however improbable, must be the truth' (A. Conan Doyle, 1885)*

At present, it is unlikely that someone living in the UK would receive a medical diagnosis of Electromagnetic Hypersensitivity (EHS). In its 2005 ‘Backgrounder' (1), the World Health Organization (WHO) recognizes the condition, and the symptoms that EHS individuals associate with Electromagnetic Field (EMF) exposures. WHO accepts that the symptoms are certainly real, and that they can be disabling for some.

However, it says that there is no evidence linking EHS to EMF exposure, and that it may be linked to other environmental factors, stress or to a pre-existing psychiatric condition. It says that EHS is not a medical diagnosis, and that treatment should not focus on reducing EMF exposures, as symptoms could be caused by worrying about EMFs. This view finds support (2, 3), although not universally (4, 5), and Dieudonné finds no hypothesis totally satisfying (6).

The condition is not coded in the WHO's International Classification of Diseases [ICD; (7)], despite calls for its inclusion (8–10). Individuals may therefore not be diagnosed due to the official position on EHS, plus a lack of consensus regarding biomarkers (11) and controversy over the etiology. In addition, advice on potentially beneficial treatments (12, 13) may not be provided.

Lacking a formal medical diagnosis, an EHS individual might self-diagnose their own condition. If they are aware of EMFs, they would use one or more EMF detection meters to see what they are being exposed to, where, how often and at what levels, and then correlate these exposures with their symptoms.

Due to a widespread lack of recognition and support (14), an EHS individual may encounter stigma, disbelief and ridicule. They may stop working, and have to rely on alternative sources of income. Their quality of life may be harmed. They may flee their home, move to an area with lower EMFs, and limit time spent in places where EMF levels are high. They may have to sacrifice their careers and interests. Their relationships may suffer. They may experience discrimination. They may lose faith in the authorities, and in the healthcare system.

Having been EHS since 2007, I know about these challenges, and this article is a non-academic account of how I eventually self-diagnosed EHS. I cannot prove a link between my condition and EMFs, and I cannot explain what EHS is, and why some people seem to be susceptible. Nevertheless, I hope that my first-hand account can complement ongoing scientific research.

## A mystery

I used to be fit, healthy and active. I spent lots of time outdoors, played sport, and traveled fairly extensively. Ironically—as things turned out—I worked in the technology sector, developing computer systems. Despite my occupation, I was ambivalent about technology. I did use a mobile phone and a DECT cordless phone, but I did not have Wi-Fi, or any other wireless devices.

My first mobile phone was very bulky, and my second was a sleeker model that was advertised as being 2.5 G. I didn't know or care what that meant; I just used it for calls, texts and for taking the occasional photo. I didn't know how wireless technologies worked, and I had never heard of EMFs.

In 2007, I suddenly started experiencing symptoms. Intermittent at first, they became increasingly frequent and severe. Initially, they were mainly chronic headaches, dizziness and vertigo, but other symptoms appeared later. Now I realize that they were classic EHS symptoms (15–17). At the time, I had no idea what was happening. I managed to continue working, and because the symptoms were intermittent, I took lots of over-the-counter painkillers to deal with them, rather than seeking medical advice.

In 2008, I was using a laptop for extended periods. My symptoms worsened considerably, and eventually they became so bad that I checked myself into a hospital for tests. These revealed nothing of significance, and I was prescribed some pills, which made no difference.

By then, I was nearly at the end of the contract. This was fortunate, as I could no longer use my laptop due to my symptoms, and one of my colleagues had to complete my work. I returned home, and had a break from work for a while.

Over the next 2 years, I carried on working, still taking lots of painkillers. One day, after a sleepless night, I was in a bad state. I managed to drive to work, but while I was there, my symptoms became so severe that I left the office. That was the last day that I ever worked.

I then spent 3 years trying to get a diagnosis from the National Health Service (NHS). Test results weren't significant, and—once serious diseases had been ruled out—my case was not treated as urgent. I had to keep asking for referrals to specialists who might be able to help.

The treatment that I received from the NHS consisted of a number of different prescription drugs (tricyclic antidepressants, anticonvulsants and anti-vertigo preparations), all of which made me much worse; some sessions with a cognitive behavioral therapist and then a psychologist, with no improvement seen, and some physiotherapy.

I became disillusioned with the NHS. Instead, I spent a fortune on private consultations, scans, tests and various complementary treatments, but the tests didn't find anything significant, and the treatments were ineffective, as I didn't know what I was trying to treat.

## The improbable truth

I read many medical and complementary healthcare books, looking for answers. One day, a website algorithm recommended a book about EMFs and EHS—subjects that I hadn't heard about before. This was in 2012; five years after my condition started

After I had read the book, I was skeptical, but I wanted to test if I could be reacting to EMFs. I stopped using my DECT cordless phone, and I switched my mobile phone off when not in use. This made no difference, and so I set aside that theory, and continued the search for answers elsewhere.

I discovered ‘Energy Medicine,' and received two different electrotherapy treatments. I'd originally contacted the practitioner about receiving PEMF (Pulsed Electromagnetic Field) therapy, and he'd suggested that I also have a second treatment, which uses a hand-held device that sends extremely low frequency electronic impulses via the skin to the brain, with the aim of triggering the body's immune system to kick in.

The practitioner would apply this device onto my bare back, and although I could tolerate it at first, he had to use increasingly sensitive settings as the sessions progressed. It felt very painful, like having acid applied to my back, and my condition got noticeably worse.

This extremely negative reaction to electrotherapy, which was supposed to make me better, not worse, made me reconsider Electromagnetic Hypersensitivity. In 2013, I bought a Cornet ED78S meter, which detects Radiofrequency Radiation (RFR), and Extremely Low Frequency (ELF) Magnetic Fields (MF) which are emitted by electrical items, wiring and infrastructure. When I used this meter for the first time in my home, it was revelatory.

I then bought a Stetzer ‘Dirty Electricity' (DE) meter, which measures the erratic electrical ‘noise' that emanates from household wiring, and, some years later, I bought a Cornet ED88T, which detects ELF Electric Fields (EF), as well as RF and MF.

(Note: These events happened 10 years ago; I wasn't recording the EMF levels, and so I cannot include specific details here. Also, I now know that I was concurrently exposed to a complex mix of different EMF frequencies, types and intensities, with unknown synergies.)

I had previously assumed that my phones were my only EMF sources. However, the meters revealed much more:

- Wi-Fi, DECT cordless phone, microwave oven and mobile phone RFR was entering from adjoining properties.
- My electricity meter was emitting RFR throughout the house.
- The RFR from mobile phone antennas on top of a block of flats was penetrating my house, particularly upstairs. I hadn't even noticed these structures before.
- The MF and DE levels in the house were generally high.

These meters explained to me why I felt so much worse in certain parts of the house, where the EMFs were at their highest, and the Cornet meter showed how much RFR I was exposed to from my mobile phone, my electricity meter, outside sources and my neighbors' wireless devices. I was also shocked when I plugged my DECT cordless phone back in, and measured the RFR levels.

What I had found out was enough for me to self-diagnose EHS, 6 years after my condition had started. None of the health practitioners that I had seen had ever mentioned this condition to me, and so I was left to discover it for myself.

My primary ‘treatment' became (and remains) the reduction of my exposure to all types of artificial EMFs. The development of my condition, the challenges that I faced, and my symptom management, correlate with the experiences of other EHS individuals (18). My wife is also EHS, so this provides another basis for comparison.

## Is the problem just wireless technologies?

As well as being affected by RFR (plus ELFs and DE), these natural phenomena can also exacerbate my symptoms:

- Strong winds, storms, precipitation, fog
- Coronal mass ejections/geomagnetic disturbances
- Waxing Gibbous moon phase

Other stress factors that can exacerbate my symptoms include: excess caffeine, prescription drugs, poor quality sleep, stress or trauma, chemical fumes, air pollution and insect stings.

## Limiting exposures

I can decide how much coffee to drink. Similarly, some EMF exposures in my home are also under my control. For example, I don't have any wireless devices. Even the ELFs and DE can be reduced, by switching the power off at the consumer unit overnight, and by unplugging electrical appliances when not in use. I can also limit time spent on the computer, or in making phone calls.

Artificial EMFs from external sources are much harder to deal with, because I have found that EMF shielding products can ‘fix' one problem, while introducing others. I therefore have very little control over EMFs originating from neighboring properties, mobile phone masts, Tetra emergency services masts, electrical distribution infrastructure and so on.

I can be in public places with relatively high EMF levels, but I limit my time in them. The problem now is that the whole environment is saturated with artificial EMFs, so for EHS people like me, there are very few low EMF places left to go to.

## Discussion

In this article, I've briefly described my EHS self-diagnosis. Based upon my lengthy experience with the condition, I've reached a number of conclusions:

- EHS is a neglected public health issue.
- The prevalence of EHS is unknown.
- The healthcare system fails EHS individuals.
- ICNIRP's EMF limits do not protect EHS individuals.
- EHS is multi-factored, and overlaps with other chronic health conditions.
- Many EHS studies have significant limitations.
- Support for sufferers is poor, to non-existent.
- EHS results in significant societal, economic and personal costs.

WHO's position, and the lack of an ICD code, leaves EHS individuals in medical limbo. This places an extra burden on the healthcare system, because the lack of a timely diagnosis can lead to many unnecessary consultations, tests, scans and treatments being carried out. There is therefore an urgent need for increased awareness among health professionals (19, 20).

There are divergent views on treating EHS, but I believe that EMF reduction is fundamental. Had I been diagnosed with EHS, I would have started reducing my exposure to EMFs much sooner, and I would not have represented such an ongoing drain on scarce NHS resources.

In my opinion, scientific research into EHS needs to be of much higher quality, and should seek to identify specific EHS biomarkers. Viable tests for these biomarkers would facilitate the identification and treatment of EHS individuals, and ultimately help us to move toward prevention of the condition.
